# In Situ Sol–Gel Synthesis of Unique Silica Structures Using Airborne Assembly: Implications for In-Air Reactive Manufacturing

**DOI:** 10.1021/acsanm.2c02683

**Published:** 2022-08-17

**Authors:** Connor R. Barker, Francesca K. Lewns, Gowsihan Poologasundarampillai, Andrew D. Ward

**Affiliations:** † Department of Earth Sciences, Royal Holloway University of London, Queens Building, Egham, Surrey TW20 0EX, U.K.; ‡ STFC, Central Laser Facility, Research Complex at Harwell, Rutherford Appleton Laboratory, Didcot, Oxfordshire, OX11 0FA, U.K.; § School of Dentistry, University of Birmingham, 5 Mill Pool Way, Birmingham, B5 7EG, U.K.

**Keywords:** sol−gel, silica, Raman spectroscopy, Mie spectroscopy, FIB−SEM, aerosol, optical trapping, droplet deposition

## Abstract

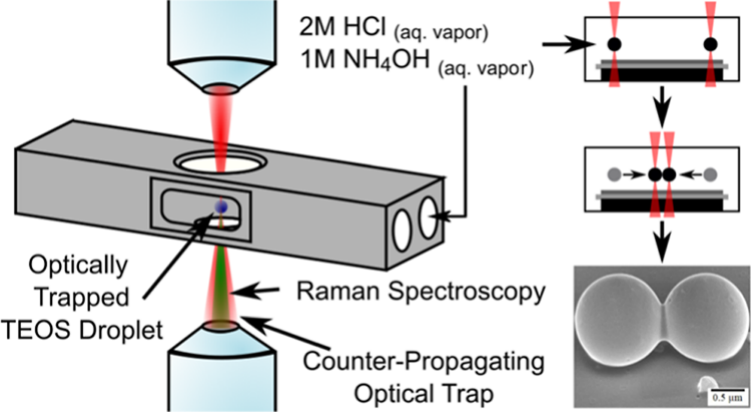

Optical trapping enables the real-time manipulation and observation of morphological evolution of individual particles during reaction chemistry. Here, optical trapping was used in combination with Raman spectroscopy to conduct airborne assembly and kinetic experiments. Micro-droplets of alkoxysilane were levitated in air prior to undergoing either acid- or base-catalyzed sol–gel reaction chemistry to form silica particles. The evolution of the reaction was monitored in real-time; Raman and Mie spectroscopies confirmed the in situ formation of silica particles from alkoxysilane droplets as the product of successive hydrolysis and condensation reactions, with faster reaction kinetics in acid catalysis. Hydrolysis and condensation were accompanied by a reduction in droplet volume and silica formation. Two airborne particles undergoing solidification could be assembled into unique 3D structures such as dumb-bell shapes by manipulating a controlled collision. Our results provide a pipeline combining spectroscopy with optical microscopy and nanoscale FIB–SEM imaging to enable chemical and structural insights, with the opportunity to apply this methodology to probe structure formation during reactive inkjet printing.

## Introduction

In recent years, additive manufacturing (AM), otherwise known as three-dimensional printing (3DP), has experienced rapid interest and development, in particular, in high-value manufacturing. Implants and scaffolds for tissue engineering, regenerative medicine, in vitro disease modeling, and drug development are a few examples of areas that have, and are, benefiting from the strong potential of AM for biomedical applications.^1,2^ Inkjet printing is one of the frontrunners due to its high resolution (sub-micron) and speed.^3−6^ Advances in droplet delivery and fabrication have enabled reactive and micro-reactive inkjet printing of multi-material complex structures.^7,8^

Inkjet printing requires an ink with a viscosity in the range of 3.5–30 mPa s^–1^^5,9^ to undergo sol–gel transition upon ejection or to rapidly cure on the platform. Thus, a large proportion of biomedical materials are not suitable for inkjet printing. Exploiting the sol–gel transition of alkoxysilanes within inkjet printing^10−13^ provides a huge potential for biomaterial applications, as low viscosity sols can be reacted in situ to form highly condensed structures alone or together with biopolymers to result in highly porous materials.^14−16^ However, insights on the in-air reactivity of alkoxysilanes are scarce due to technical difficulties in performing real-time spectroscopic measurements on droplets that are suspended for a long period of time. Exposure of tetraethyl orthosilicate (TEOS), the most commonly used alkoxysilane in sol–gel processing, to an acidic/basic environment will also result in sol–gel transition, yielding a stable and highly elastic silica-based gel.^17^ These materials have good potential for reactive jetting printing; however, clogging of colloidal particles can present as a problem during printing, and research has suggested that silica sols with a pH of 3.1 provide optimal printing behavior.^18^ Consideration of the complex reaction mechanisms and kinetics is required, as these are influenced by the catalyst used, for example, an acid or a base. How this choice of catalyst impacts the evolution of silica structures during jetting needs to be characterized and compared to conventional bulk reactions.

Optical trapping using laser beams has been previously used to manipulate aerosol droplets and study reaction behavior.^19,20^ Here, the aim was to investigate the reactivity of optically trapped TEOS droplets and to assess whether the reacted droplets could be structured into novel morphologies through controlled collision using multiple optical traps to imitate the jetting process. To achieve this aim, it was necessary to study sol–gel reactions within individual aerosol droplets, focusing on acidic and basic catalyses, the time taken for gelation to occur under specific catalytic conditions, and the changes in the droplet volume as the sol–gel reaction proceeds. The reaction chemistry was followed with Raman spectroscopy and Mie scattering within the Raman signal.^21^ The resulting particles were deposited on a substrate for further imaging of nanoscale structures by focused ion-beam–scanning electron microscopy (FIB–SEM).

## Experimental Section

### Optical Trapping of Single TEOS Droplets

**Figure 1:**
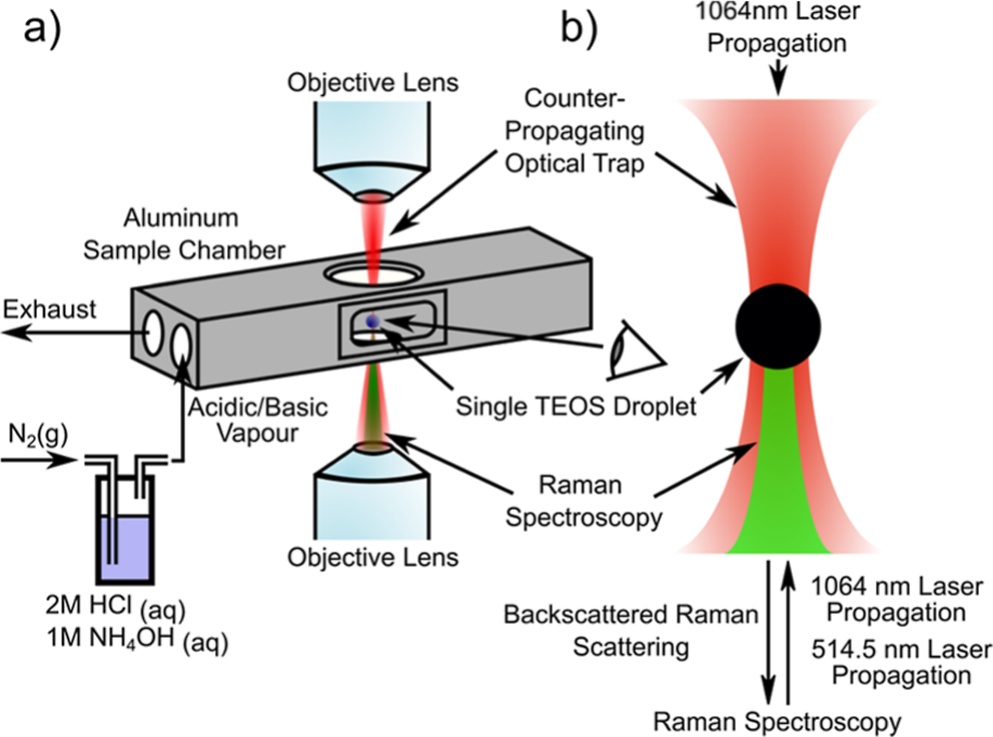
(a) Apparatus used to trap, observe, and assemble silica structures. (b) Diagram of the optical trapping and Raman spectroscopy laser beams at the foci of the laser beams.

An ultrasonic nebulizer was used to produce aerosol droplets of TEOS. These were delivered through 6 mm PTFE tubing to an aluminum sample chamber (Figure 1a) and trapped using an infrared laser beam (1064 nm) that was focused through opposing objective lenses to form a stable optical trap.^22,23^ To ensure efficient delivery of a concentrated catalytic vapor to the sample chamber, nitrogen gas was passed through a bubbler containing 2 M hydrochloric acid or 1 M ammonia for acidic or basic catalysis, respectively. The 2 M HCl and 1 M NH_4_OH aqueous solutions had partial pressures of 2.0 × 10^–4^ and 5.2 × 10^–2^ kPa, respectively.^24^ Flowing nitrogen through the aqueous catalysts in this way resulted in gaseous concentrations of 4.7 × 10^13^ and 1.2 × 10^16^ molecules cm^–3^ for the acidic and basic catalytic vapors, respectively,^25^ and ensured that the sample chamber environment was saturated with the gaseous catalytic vapor. Sol–gel reactions were measured in real-time with Raman spectroscopy using a co-axial Ar-ion laser beam (514.5 nm) focused through the lower objective lens, and the backscattered Raman scattering was collected through the same objective lens (Figure 1b). The experimental method is detailed fully in the Supporting Information.

### Dual-Particle Analysis and Deposition

**Figure 2:**
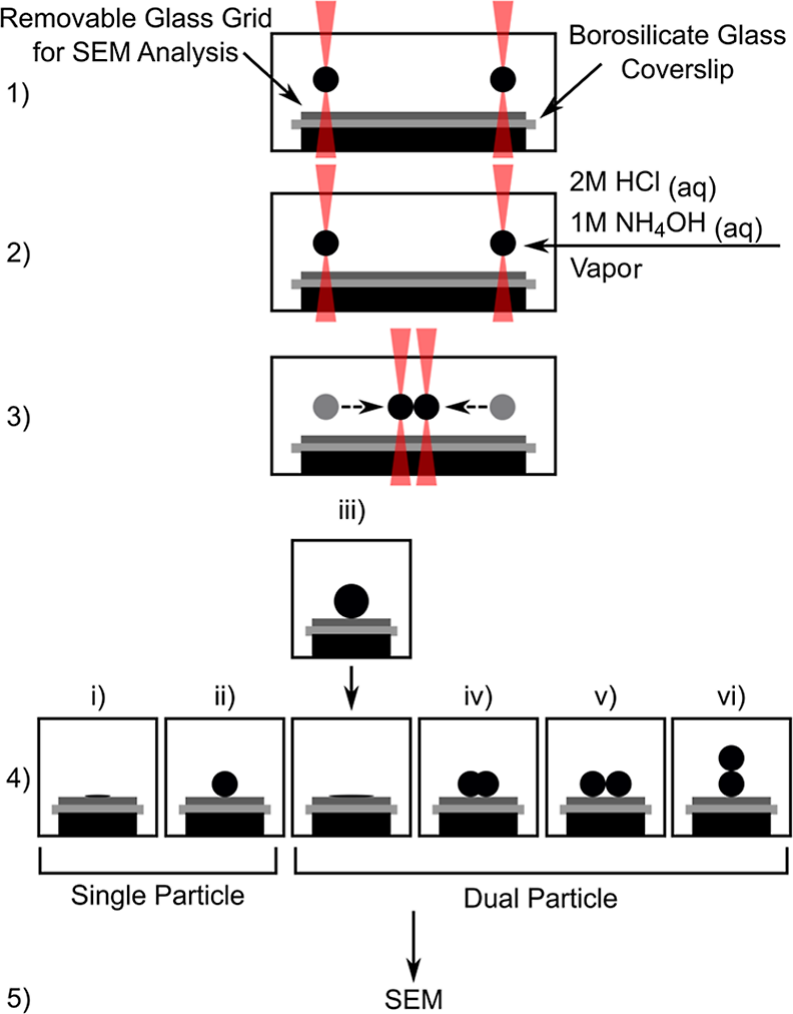
Overview of the droplet collision, coalescence, and deposition processes for multiple droplets and the resulting morphologies. Process of droplet deposition is outlined, including (1) trapping, (2) acidic/basic hydrolysis, (3) collision of droplets, (4) deposition of droplets, and (5) SEM analysis.

An acousto-optic deflector was placed in the optical path of each trapping laser to produce two time-shared optical traps at the sample plane, with a modulation time of several milliseconds. The separation of the two optical traps was calibrated and controlled externally using LabVIEW software. Two TEOS droplets were trapped simultaneously at a large separation (schematically shown in Figure 2), reacted in situ, and then brought together slowly until contact, leading to hard sphere-on-sphere contact, coalescence, or novel fused dumb-bell structures with nanoscale necking from partial merging of the droplets. The experimental outcomes of deposition displayed in Figure 2 are as follows: (2i) the single droplet remains liquid and spreads onto the glass slide. (2ii) The single droplet reacts to form a solid and remains a hard sphere after deposition. (2iii) Two liquid droplets coalesce to form a larger droplet, which spreads onto the coverslip upon deposition. (2iv) Partial reaction of two droplets forms a partially merged structure. (2v/2vi) Two solidified spheres collide and no coalescence occurs, leading to a two-sphere structure which remains horizontal or rotates in the optical trap to a vertical alignment.

**Figure 3:**
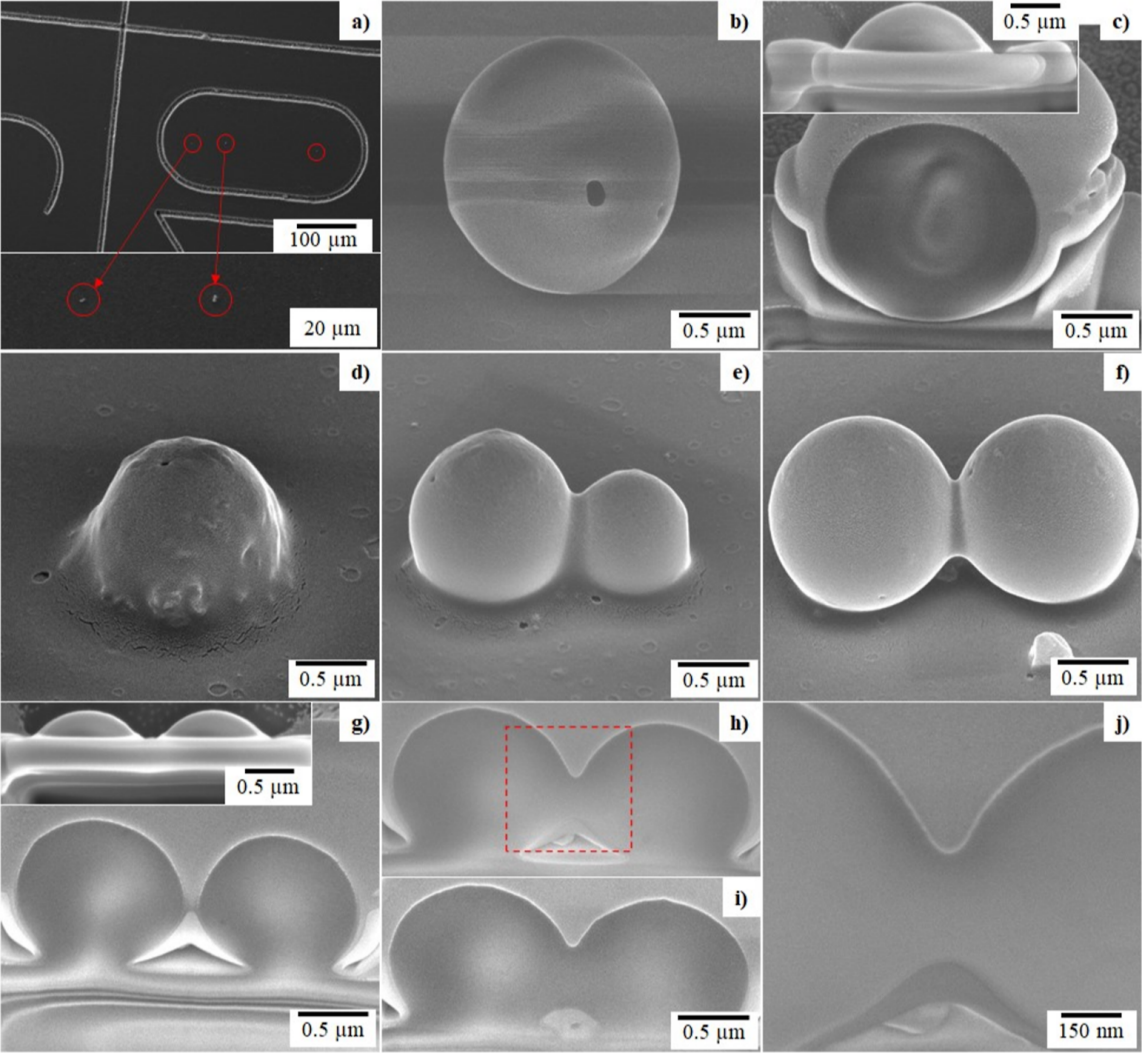
Morphology of the silica particles produced and imaged by SEM. (a) Glass slide used for particle deposition. (b) Morphology of a single silica particle reacted in air for 4 h under 2 M HCl vapor, including the (c) cross-section and top-view (c) inset) of the particle after FIB milling to a certain depth. (d–j) Silica particles formed after two TEOS droplets were reacted in air under 1 M NH_4_OH vapor for (d) 5, (e) 6, and (f) 7 min and subsequently collided together for coalescence. (g–i) Cross-sections and top-view (g) inset of coalesced particles in (f) after FIB milling to various depths. (j) High magnification image of the boxed region in (h).

Particles and merged structures were collected by raising the sample cell to the levitated droplet until surface contact. The collected particles were 1–2 μm in size and spherical. These were deposited at known locations onto a glass slide on the coverslip, defined by imprinted grid patterns (Figure 3a). FIB milling was employed to mill away particles, and cross-sectional images were formed with SEM. The insets of Figure 3c,g show the top-view of the silica particles in Figure 3b,f after part of the particles have been milled away.

## Results and Discussion

### Particle Morphology

TEOS droplets were reacted for 5–240 min, resulting in particles of various morphologies (Figures 2 and 3). The FIB-milled cross-sections of particles imaged with an electron beam showed a uniform nanostructure both compositionally and architecturally at the highest magnification used (Figure 3j). The degree of coalescence and thus the structures formed when multiple droplets were brought together were found to be dependent on the reaction time (Figure 3d–f).

Particles synthesized under basic catalysis were found to completely coalesce, forming a single larger particle when reacted for 5 min or less (Figure 3d). Following deposition on the glass slide, this particle had partially flowed, fusing with the glass slide. Droplets that reacted for 6 (Figure 3e) or 7 min (Figure 3f) underwent partial coalescence, leading to a dumb-bell shape with a smaller neck at the longer reaction time. These particles had also partially flowed over the glass slide fusing with it. Cross-sections produced by FIB milling and SEM imaging of the coalesced particle in Figure 3f are shown in g–j. As with the single particle (Figure 3c), the coalesced particle also exhibited a homogeneous bulk, and no voids were observed in the magnification image.

Furthermore, a magnified image of the neck region shows no distinct boundary (Figure 3j). This suggests that the particles were bonded together strongly via mixing of the two particles at the neck, forming a single structure. All the particles were observed to have at least one pore or hole on their surface regardless of the reaction time, whether for 5 min or 4 h. The pore was typically between 10 and 50 nm in radius and is speculated to arise from refocus of the trapping laser refracted at the front of the spherical particle onto the rear surface of the particle.

### Sol–Gel Reaction Kinetics

During a sol–gel reaction, an alkoxysilane, such as TEOS, is converted into its oxide, silica (SiO_2_). The chemical structure of the oxide evolves as the product of successive hydrolysis and condensation reactions (Figure 6a).^26^ Reaction rates are influenced by the type of catalyst and available water as well as other factors.^27,28^ The moles of water per moles of alkoxide, known as the *R*-ratio, necessary to complete the polycondensation of silanes is dependent on the number of hydrolyzable (alkoxide) groups in the silane molecule. An R-ratio of approximately 2 is required for the complete hydrolysis of TEOS.^29^ An increased R-ratio from 2 increases the hydrolysis rate up to a threshold, after which it begins to inhibit the reaction.^29,30^ This behavior has been related to the solubility of the alkoxysilanes.^30^ For acidic catalysis, the degree of hydrolysis and condensation is dependent upon the availability of water; however for basic catalysis or neutral systems, the water content does not significantly affect the final structure of the product.^31−33^ In acid-catalyzed reactions of TEOS with a low *R*-ratio,^34^ monomeric silanol species hydrolyze and condense to form linear chain-like structures rather than colloidal particles. These chain structures are highly entangled and undergo gelation through crosslinking between overlapping chains. Contrastingly, basic catalysis, as in the Stöber process,^35^ results in a high degree of branching and the formation of large individual clusters (∼200 Å) that are dense. These can then link together to form a gel. Thus, the catalyst type, *R*-ratio, and pH of the reaction medium can influence polymerization kinetics.^29^

**Figure 4:**
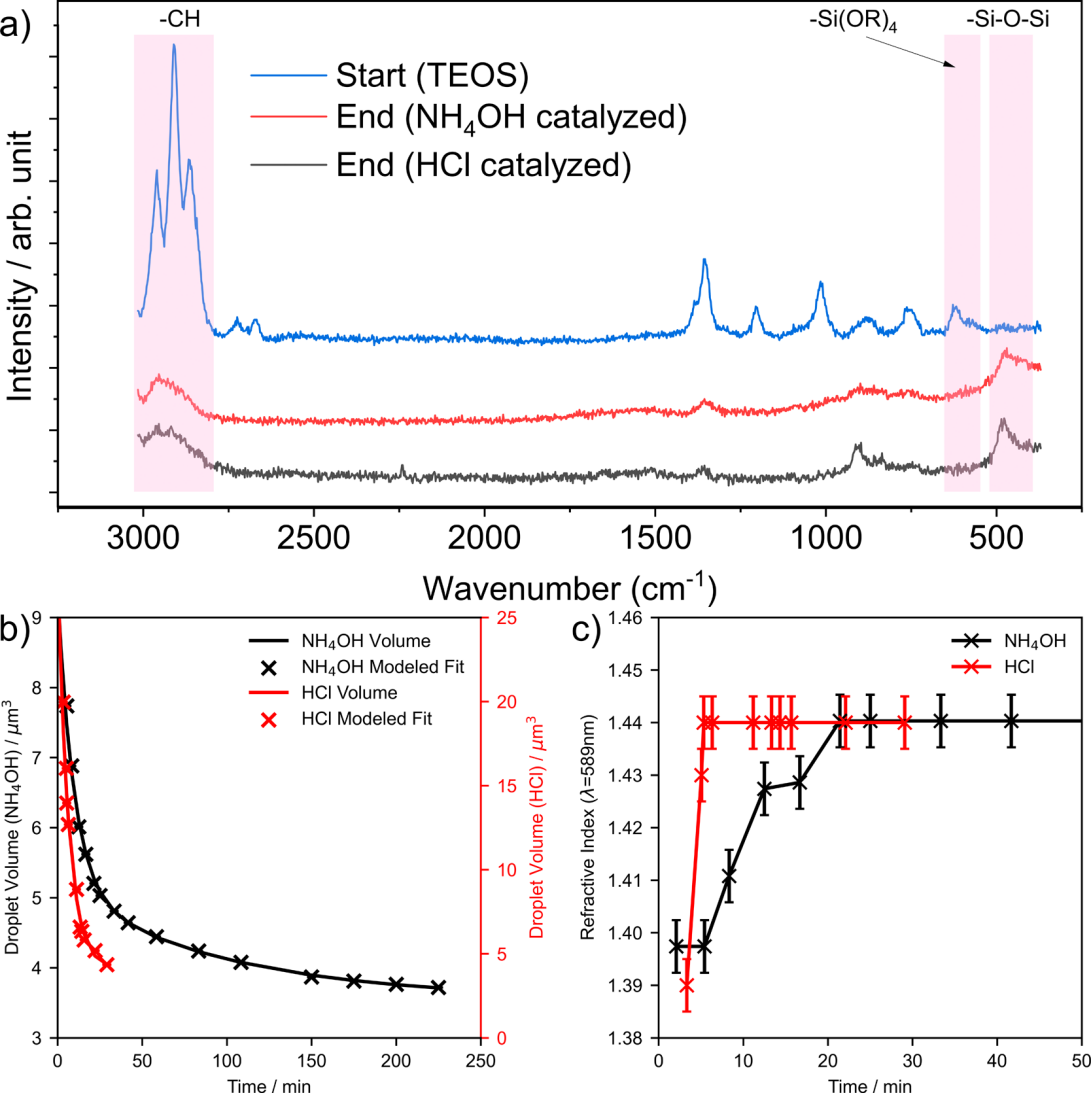
(a) Initial and final Raman spectra of the in situ sol–gel formation of a silica-like particle from a TEOS droplet catalyzed with NH_4_OH and HCl. (b) Droplet volume and (c) refractive index values at 589 nm throughout the sol–gel reaction under NH_4_OH and HCl catalyses. The error bars reflect the variation of the optimum theoretical solutions while modeling each recorded spectrum; the uncertainty for volume is less than the symbol size.

**Figure 5:**
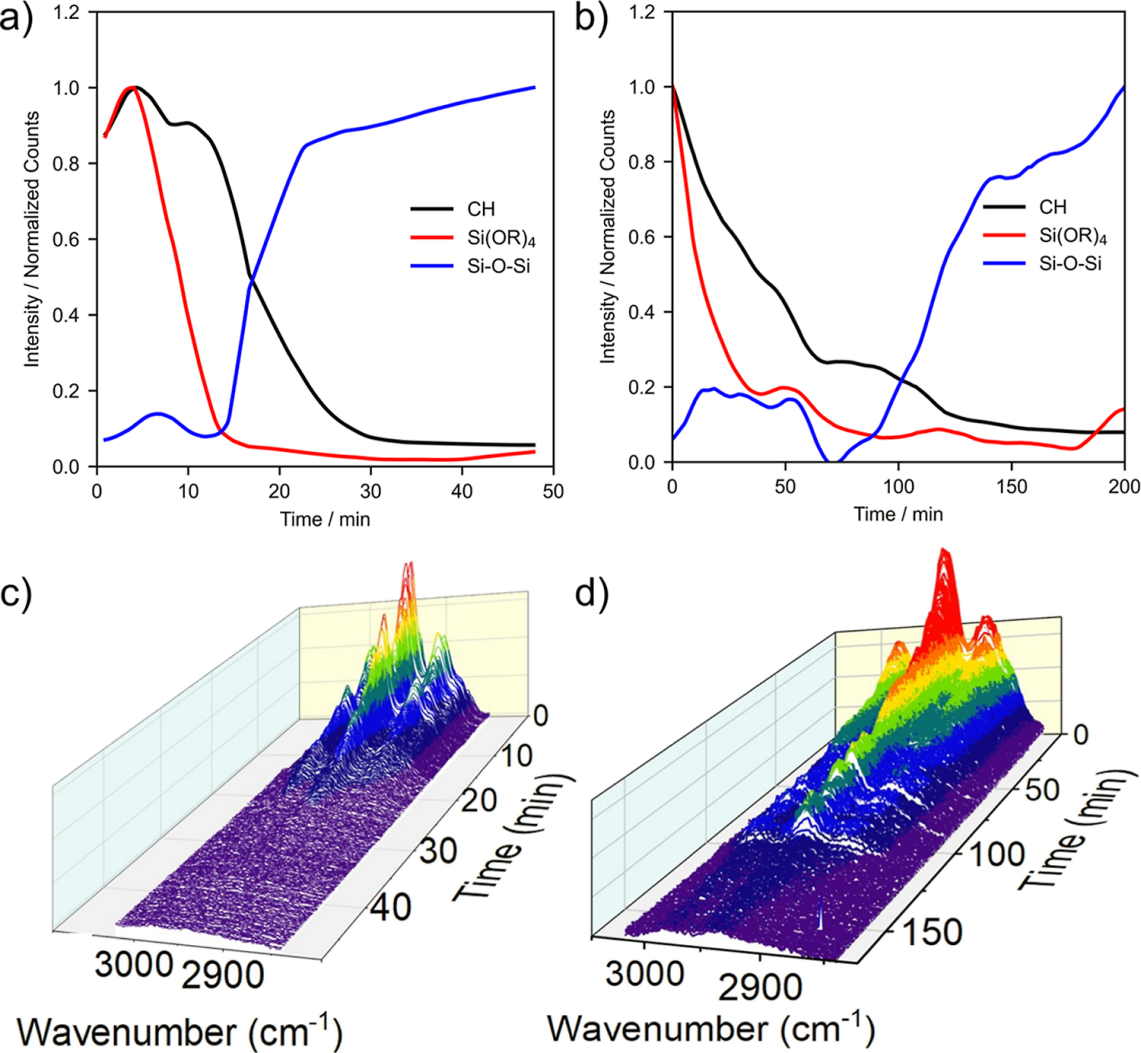
Variations in the normalized spectral intensity of CH, SiOR_4_, and Si–O–Si bands during a catalyzed sol–gel reaction of TEOS with (a) HCl and (b) NH_4_OH smoothed using an adaptive baselining technique to remove Mie resonances and normalized for comparison. Evolution of the CH vibrations in the Raman spectra throughout the sol–gel reaction, displayed as 3D waterfall plots as a function of time for reactions catalyzed by (c) HCl and (d) NH_4_OH. Similar plots for the SiOR_4_ and Si–O–Si vibrations are included in the Supporting Information.

**Figure 6:**
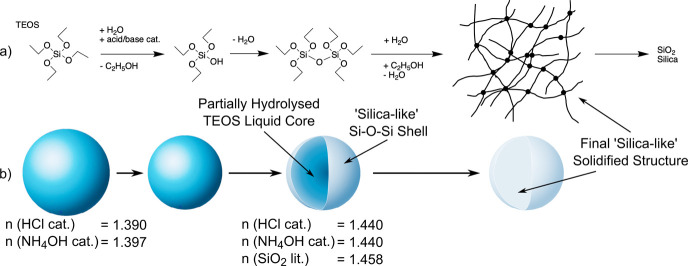
Overall sol–gel reaction of TEOS to silica, with individual reaction steps and the corresponding state of the trapped TEOS droplet transitioning to a “silica-like” Si–O–Si structure. (a) Overall sol–gel chemical reaction scheme of TEOS to silica, including an illustration of the “silica” like structure.^34^ (b) Hypothesized changes in droplet structure throughout the reaction. All refractive indices, *n*, are for λ = 589 nm.^49,50^

The Raman spectra of levitated droplets were collected with acquisition times of 2 and 5 s (Figures 4a and 5c,d). The evolution of the chemical composition of a single droplet of TEOS could be monitored in real-time, following the introduction of the catalyst and throughout the sol–gel reactions (Figures 4a, 5 and 6a). Raman spectra of TEOS droplets exposed to HCl and NH_4_OH vapor show peaks corresponding to TEOS, 2931 cm^–1^ (C–H) and 653 cm^–1^ (Si-(OR)_4_) (Figure 4a), which decrease in intensity during the sol–gel reaction. A broad peak at 499 cm^–1^ (Si–O–Si) is then observed, indicating the in situ formation of silica within the trapped droplet. The product from either acidic or basic catalysis appears to be similar when comparing the final Raman spectra.

Figure 5a shows plots of normalized intensity of the C–H, Si(OR)_4_, and Si–O–Si vibrations as a function of time for an acid-catalyzed sol–gel reaction. The decrease in the C–H and Si(OR)_4_ vibrations indicates that TEOS was rapidly hydrolyzed,^36^ resulting in the loss of material, namely ethanol, through evaporation from the droplet (see also Figures 4b and 6). The disappearance of the Si(OR)_4_ vibrations after 15 min may also indicate that nearly all the molecules of the initial TEOS reactant have been hydrolyzed, although, at this point, most of the C–H material is still within the droplet. Si–O–Si bond formation is only seen at low levels (<10% of the final product) during the first 10 min. The formation of Si–O–Si bonds through condensation progresses steadily once hydrolysis of TEOS is complete and continues until extended silica structures are formed (Figure 6a). Some hydrocarbon material appears to be retained in the final product.

The rate of hydrolysis under basic conditions was significantly slower, and the onset of silica formation occurred at around 70 min [Figure 5b]. The single droplet reactions follow a similar behavior with respect to pH to that reported previously.^29^ Thus, in acidic medium, hydrolysis is fast and condensation is slow, meaning condensation forms the rate-limiting step. Conversely, in basic medium, hydrolysis is slow and condensation is fast, and therefore, hydrolysis is the rate-limiting step.

### Evolution of Droplet Size and Refractive Index

A broader series of peaks were observed in the Raman signal that shifted in wavenumber as the experimental run progressed. These peaks are Mie resonances arising from the weak spontaneous scattering of light across the Raman spectral range.^37−39^ Mie scattering spectra were extracted using temporal filtering (i.e., the Mie spectral positions change with time, while Raman spectral positions are essentially fixed for these studies). The Supporting Information includes a more detailed description of the temporal filtering process and an image of the Mie resonance shifting with time.

The Mie resonances were compared to a theoretical Mie scattering model^40^ to retrieve the refractive index and volume of the droplet over the course of the reaction.^19,41−45^Figure 4b,c shows the evolution of the volume and refractive index (at 589 nm), respectively, for a TEOS particle after exposure to acidic (HCl) and basic (NH_4_OH) catalysts.

The decrease in droplet volume over time can be described by two simultaneous first-order equations
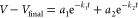
1

The *a*_1_ and *a*_2_ parameters (Table 1) correspond to an initial volume *V*_0_ for each first-order equation, and the constants *k*_1_ and *k*_2_ (Table 1) describe the rate of change of the droplet volume. The values of *k*_1_ can be used to compare the relative reaction rates of the HCl- and NH_4_OH-catalyzed reactions. The fitting process for these equations is detailed in the Supporting Information.

**Table 1:** Rate Constants Describing the Decrease in Droplet Volume During the Sol–Gel Reaction

	NH_4_OH	HCl
*a*_1_/μm^3^	4.25	24.16
*a*_2_/μm^3^	1.59	N/A
*k*_1_/s^–1^	1.75 × 10^–3^	2.63 × 10^–3^
*k*_2_/s^–1^	1.72 × 10^–4^	N/A
*V*_i_/%	55	57

The initial rapid loss in the volume of the droplet (Figure 4b) indicates that the particle undergoes a fast reaction step, described by k_1_. This initial reaction step is completed in <∼5 and ∼21 min for the HCl and NH_4_OH catalysts, respectively, after which a slower reaction step, described by *k*_2_, dominates. Thereafter, the droplet size gradually reduces until the reaction is complete. The second component *k*_2_ is approximately an order of magnitude slower than *k*_1_ under basic catalysis.

The initial volume decrease is consistent with the observation of nearly total TEOS hydrolysis from the simultaneous Raman spectroscopy measurements (Figure 6). Acidic catalysis was found to be ∼1.5 and ∼7.2 times faster than basic catalysis for the rate of loss of droplet volume, *k*_1_. Therefore, the volume changes also agree well with the literature that acidic catalysis causes rapid hydrolysis of TEOS, with considerably slower hydrolysis under basic conditions.^29^

The slower component *k*_2_ describes the slow loss of droplet volume which dominates toward the end of the reaction and can be attributed to an increasing density within the droplet. As successive condensation reactions occur, the droplet composition tends toward the final “silica-like” structure (Figure 6). This is observed in the Raman spectra with an increase in Raman intensity at ∼499 cm^–1^ (Si–O–Si). The order of the condensation reaction step varies in the literature from the first to the fifth order^46,47^ and has been shown to be of first order under the addition of salt as a catalyst. In this case, the ionic strength of the salt determines whether it acts as an acid or a base.^29,48^ In the experiments performed here, the relative values of *k*_1_ and *k*_2_ for basic catalysis (Table 1) indicate that condensation proceeds at approximately an order-of-magnitude slower rate than hydrolysis under acidic catalysis.

The calculated increase in refractive index at 589 nm (Figures 4c and 6b) appears to be rapid for both HCl and NH_4_OH catalysts. The refractive index is constant before and after this increase, and so Figure 4c shows only the first 50 min. The timescale for the refractive index to increase is comparable to the time at which evaporation is complete and gelation begins to dominate, as inferred from the droplet size evolution. It is also comparable to the onset of gelation behavior determined by contact of droplet surfaces. The droplet volume *V*_i_ (Table 1) at this point was calculated as a percentage of the initial volume *V*_0_, determined as the summation of *a*_1_, *a*_2,_ and the final volume. This intermediate volume, *V*_i_, is comparable for both catalytic conditions but is reached in a shorter timescale for catalysis with HCl. Additionally, SEM imaging of TEOS droplets that had been reacted in a catalyzed sol–gel reaction (Figure 3) demonstrated that both catalytic conditions resulted in solid particles. The comparable loss in droplet volume and similar final morphologies indicate that the physical changes to the droplet (Figure 6b) occur through the same mechanism for both HCl and NH_4_OH catalysts but at differing reaction rates.

The refractive indices of silica and ethanol are 1.458^49^ and 1.361,^50^ respectively, at 589 nm, and so an increase in refractive index, with a simultaneous rapid loss in droplet volume, is consistent with the loss of ethanol and the formation of Si–O–Si bonds to yield a silica–enriched droplet. It is hypothesized that the sol–gel reaction occurs from the outer parts of the aerosol droplet through a heterogeneous chemical reaction.^51^ The rapid change in the refractive index is then due to the initial formation at the droplet surface of a “silica-like” shell enriched in Si–O–Si groups, causing a large increase in droplet surface density (Figure 6). This mechanism may explain why the reaction does not go to completion, as evidenced by the −CH peak remaining in the Raman spectra after the experimental run is complete (Figure 4a). The formation of the “silica-like” shell may inhibit further access of the catalyst vapor to the center of the droplet, where partially hydrolyzed TEOS remains (Figure 6), and prevent additional loss of ethanol through evaporation. The basic catalysis of TEOS creates a highly branched and dense polymer network compared to the less dense linear chain-like polymer network produced in acidic catalysis.^29,34^ Therefore, the structure of the “silica-like” shell is likely to be dependent on the catalytic conditions, with basic catalysis forming a denser shell compared to acidic catalysis. This would further explain the slower kinetics observed in basic catalysis, where the denser shell further inhibits the access of the NH_4_OH vapor to the droplet center and reduces the loss of ethanol through evaporation. The subsequent slow loss of droplet volume after the shell formation is attributed to an increasing droplet density as condensation polymerization occurs. However, the formation of a solid silica shell may physically limit further size changes during a continued reaction. Increased porosity inside the droplet would indicate such behavior; however, this was not observed in the SEM images. It is noted the final refractive index of the silica product is lower than that reported in the literature for bulk silica.

## Conclusions

In summary, optically trapped aerosol droplets of TEOS were isolated and studied to determine the formation of bespoke silica structures in situ and the effect of acidic and basic catalyses on hydrolysis and condensation reactions. The analysis of the chemical structure by Raman spectroscopy and that of the refractive index and volume of the droplet by Mie spectroscopy identified differences in the reaction kinetics dependent on the catalytic conditions, with acidic catalysis found to result in much faster hydrolysis than in basic conditions. Additionally, nanoscale FIB–SEM imaging of the solidified droplets after the reaction was complete showed no obvious difference in the end structure for both acidic and basic catalyses. Finally, FIB–SEM imaging of multiple merged droplets showed that the degree of coalescence was dependent upon the reaction time. Complete particle coalescence to form a single large particle was observed when TEOS particles were reacted for less than 5 min under basic conditions (1 M NH_4_OH). Thus, for in-air reactive manufacturing processes under these conditions, this work identifies an optimum sol–gel reaction time under basic conditions of 6–7 min to allow for partial coalescence of silica particles. Our results provide a novel pipeline combining spectroscopy in the form of Mie and Raman, along with optical and FIB–SEM imaging, to enable chemical and structural insights on airborne particles. The described optical trapping technology has the potential to further increase the understanding of existing inkjet printing processes. The droplet size ranges and timescales are sufficiently flexible to follow a wide range of conditions that could potentially lead to a significantly higher resolution in inkjet printing. In addition, the demonstrated multi-particle approach controlled by laser beams has the potential to be highly scalable and enable templating of 2D and 3D structures in situ prior to surface deposition. This provides an opportunity to apply the technique to further fields where imaging of aerosols would be beneficial. The authors have previously published studies on respiratory pharmaceuticals,^52^ atmospheric reactive processes,^53^ and aerosol-assisted sol–gel catalyses.^39^ From an assembly perspective, there are a multitude of processes such as spray-drying or powder-coating that could be deconstructed to enable further insights—for example, understanding how nanoparticles behave in aerosol-based applications to form functional surface coatings and structures.

## Supporting Information Available

Experimental methods, sample preparation, optical trapping, dual optical trapping, Raman spectroscopy, high-magnification imaging, and data analysis (PDF)
