# Supplementary material for: In Situ Sol–Gel Synthesis of Unique Silica Structures Using Airborne Assembly: Implications for In-Air Reactive Manufacturing
Source: ACS Appl Nano Mater. 2022 Aug 17;5(8):11699–706. doi: 10.1021/acsanm.2c02683 (PMC9425431; doi:10.1021/acsanm.2c02683)
Supplement: Supplementary file 1 — an2c02683_si_001.pdf [file an2c02683_si_001.pdf]

Supporting Information

In situ Sol-Gel Synthesis of Unique Silica Structures using Airborne Assembly: Implications for In-Air Reactive Manufacturing

Connor R. Barker[1,2], Francesca K. Lewns[3], Gowsihan Poologasundarampillai[3,*] and Andrew D. Ward[2,*].

AUTHOR ADDRESS

[1] Department of Earth Sciences, Queens Building, Royal Holloway University of London, Egham, Surrey, TW20 0EX, U.K.

[2] STFC, Central Laser Facility, Research Complex at Harwell, Rutherford Appleton Laboratory, Didcot, Oxfordshire, OX11 0FA, U.K.

[3] School of Dentistry, University of Birmingham, 5 Mill Pool Way, Birmingham, B5 7EG, U.K.

CORRESPONDING AUTHOR

*E-mail: g.poologasundarampillai@bham.ac.uk (G.P.); andy.ward@stfc.ac.uk (A.D.W.).
Phone: : +441214 665114 (G.P.); +447511 047302 (A.D.W.)

Contents

CORRESPONDING AUTHOR 1
EXPERIMENTAL........ 3
SAMPLE PREPARATION........ 3
OPTICAL TRAPPING 3
BRIGHTFIELD IMAGING........ 3
DUAL OPTICAL TRAP 3
RAMAN SPECTROSCOPY 3
HIGH MAGNIFICATION IMAGING OF SYNTHESIZED PARTICLES 4
ANALYSIS OF OPTICAL PROPERTIES........ 4
MODELLING OF DROPLET VOLUME LOSS 6
REFERENCES 7

EXPERIMENTAL

Sol-gel silica precursor tetraethyl orthosilicate was combined with a 1-propanol carrier solvent, nebulized, and optically trapped as single aerosol droplets in air. The reaction chemistry of the isolated droplets during acid and base catalysed sol-gel reactions was monitored using Raman spectroscopy for timescales of several hours. The final phase of the reacted sol-gel precursors was investigated through deposition onto the coverslip and imaging with focused ion beam scanning electron microscopy (FIB-SEM). Two time-shared optical traps were created at the sample plane using acousto-optic deflectors, and multiple particles were simultaneously trapped, reacted, and collided to produce novel fused dumbbell structures via coalescence. The structures were deposited onto glass slides for subsequent imaging using FIB-SEM.

SAMPLE PREPARATION

Tetraethyl orthosilicate (TEOS) (Sigma-Aldrich) was combined with a carrier solvent, 1-propanol, which readily nebulizes for facile delivery from the nebulizer into the sample chamber. A 10:1 solvent:sample volume:volume ratio was added to the chamber of an ultrasonic nebulizer (Omron NE-U22), which delivered nebulized TEOS:1-propanol aerosol through 6 mm PTFE tubing to an inlet on the side of an aluminium sample cell located at the optical trapping plane (Figure 1 in the main text). The 1-propanol carrier solvent is either lost during transit or upon optical trapping [1], which was confirmed with Raman spectroscopy of the particle at the start of the reaction. (Figure 4a in main text). Borosilicate windows on the top, base and sides of the sample cell transmitted laser light for efficient trapping and allowed for top and side-imaging of the trapped particle to aid with particle capture and optimization of trap stability. A separate exhaust outlet provided a stable flow through the sample cell for stable trapping conditions. For acid-catalysis of the sol-gel process, nitrogen gas was passed through a bubbler containing 2M hydrochloric acid (Sigma-Aldrich) at flow rates of 50 and 100 mL min^{-1} and delivered to the aluminium cell through a separate inlet to the nebulizer. For base-catalysis, a bubbler containing 1M ammonia (Sigma-Aldrich) delivered base-saturated nitrogen to the trapped aerosol [2]. The experiments were performed in an air-conditioned laboratory, with an approximate temperature of 20°C and a relative humidity of 30%. Imaging of the silane aerosol with FIB-scanning electron microscopy post-experiment indicated the particle sizes ranged from 1-3μm diameter.

OPTICAL TRAPPING

A vertically aligned counter-propagating optical trap was created using a 1064nm Nd:YAG laser (Ventus 1064, Laser Quantum), which was split into two beams using a fibre-coupled beamsplitter. The two laser beams were fibre-coupled into separate optical systems for collimation and alignment and passed through beam expansion optics to slightly overfill the back aperture of two vertically opposing objective lenses (Mitutoyo M Plan Apo NIR 50x, 0.42 NA) [3]. Horizontal alignment of the opposing laser foci (in the x-y plane with z as the direction of laser propagation) was controlled by movement of the upper objective lens using a piezo-stage (Princeton Instruments, E501). The vertical offset (~1-5μm) between the laser foci was controlled by adjusting the height of the lower objective lens, and a stable optical trap was produced between the opposing laser foci. The laser powers were maintained at 15 and 10 mW for the downwards and upwards laser beams respectively, via adjustment of the output of the fibre-coupled beam splitter and the diode power of the laser. This ratio provided stable trapping and forced the optically trapped aerosol into the focus of the lower objective lens, for focused brightfield imaging and optimal Raman scattering intensity [4].

BRIGHTFIELD IMAGING

A white LED located above the upper objective lens illuminated the particle, and a long working distance lower objective lens (Mitutoyo Plan Apo 20x, 0.42 NA) acted as a condenser to image the particle onto a charge-coupled device (CCD) located below the lower objective lens. A dichroic mirror below the lower objective lens reflected the trapping and Raman laser beams and transmitted the visible light for imaging. A second dichroic mirror placed above the upper objective lens passed the white light and reflected laser light. A separate brightfield imaging system perpendicular to the optical trap provided side-imaging of the optical trap, which was key for locating particles within the sample cell and for lowering particles onto the lower coverslip for further analysis. Optical filters removed 1064nm laser light and 514.5nm Raman laser light from both imaging paths [4].

DUAL OPTICAL TRAP

To analyse the chemistry and coalescence of reacted sol-gel precursors, a timeshared dual optical trap was constructed using acousto-optic deflectors (AODs) located at the conjugate focal plane of the optical trap in both laser paths. AODs adjust the angle of diffraction of an incident laser beam according to the change of frequency applied to a piezo-crystal. The frequency applied to the AOD in each optical path was calibrated and modulated on a ms timescale to form two stable timeshared optical traps, with the separation and calibration controlled externally using LabVIEW. Two aerosol droplets were trapped simultaneously at a large separation distance (> 10 μm), reacted, and brought together slowly until contact, leading to either hard sphere-on-sphere contact, partial or complete coalescence. The aluminium cell was then raised, and the reacted structure was lowered onto a glass coverslip (ibidi, Cat. No: 81148). An imprinted 50 μm cell location grid on the coverslip allowed for facile location of the structure during subsequent imaging with scanning electron microscope.

RAMAN SPECTROSCOPY

The reaction chemistry of the optically trapped sol-gel precursors was monitored using Raman spectroscopy, using a 514.5 nm Ar-ion laser (Coherent Innova 300C) to excite the optically trapped aerosol [5]. The laser beam was collimated, aligned, and combined

with the lower 1064 nm laser beam optical path using a dichroic mirror (Ingcrys). The lower objective lens focused the Raman laser onto the optically trapped aerosol and collected the backscattered light from the Raman scattering. The backscattered light was separated from the optical path using a beam splitter (Semrock) and focused with a lens into the entrance slit of a spectrometer (Acton SP2500i). A diffraction grating (600 grooves cm^{-1}, 500 nm blaze) separated the scattered light over a wavenumber range of 3000-500 cm^{-1} onto a charge coupled device (Princeton Instruments Spec10). Spectra of intensity vs. wavenumber were collected for integration times of 2 and 5 seconds, with a calibrated pixel resolution of 0.064 nm $pixel^{-1}$. Plots of the Raman spectral evolution of the $Si(OR)_4$ and c) d) Si-O-Si vibrations throughout the acidic and basic catalysed reactions are included in Figure S1.

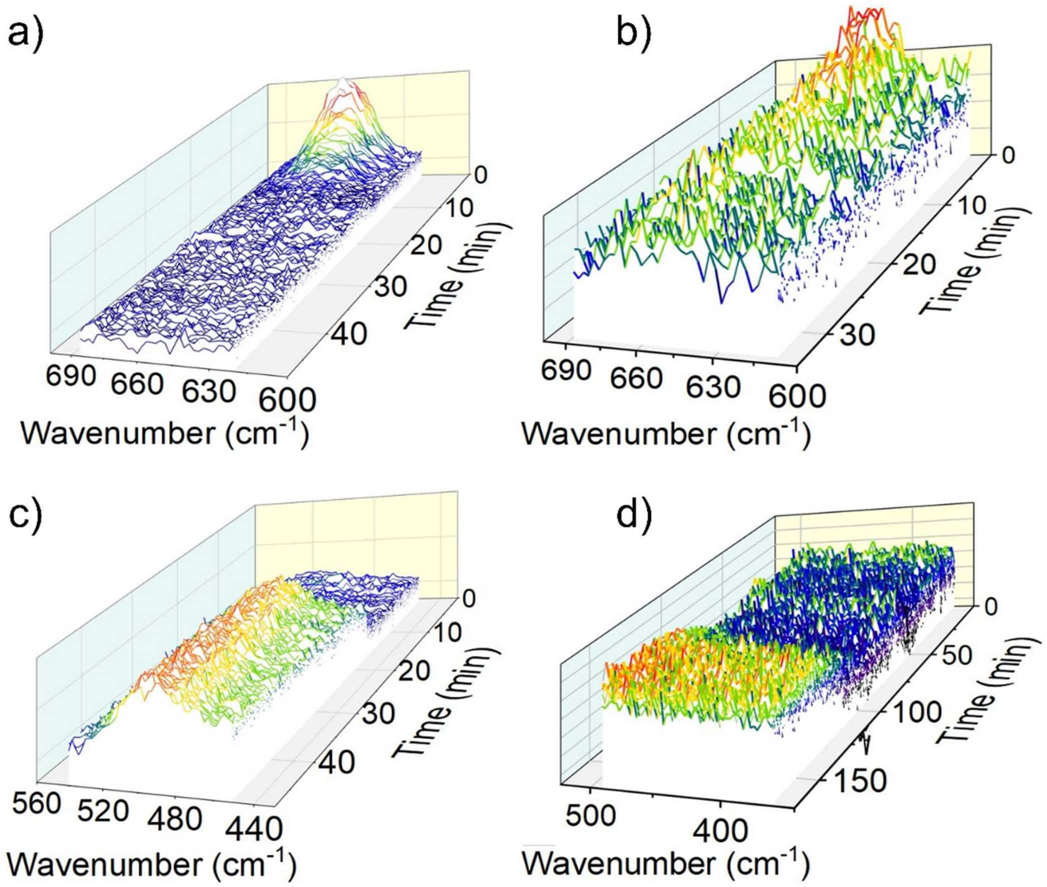

Figure S1: Evolution of the a) b) $Si(OR)_4$ and c) d) Si-O-Si vibrations in the Raman spectra throughout the sol-gel reaction, displayed as 3D waterfall plots as a function of time for reactions catalysed by a) c) HCl and b) d) NH_4OH. Plots for the CH vibrations are included in the supporting information to this text.

HIGH MAGNIFICATION IMAGING OF SYNTHESIZED PARTICLES

Samples were carbon coated prior to imaging. A dual beam FIB-SEM (FEI Nova Nanolab 600i) was used to image the particles. Cross-sections were produced using a gallium ion beam and SEM images were collected using the through lens and back scattered detectors at various accelerating voltages (1-5 kV) [6].

ANALYSIS OF OPTICAL PROPERTIES

In the collected Raman spectra, a separate broader series of peaks were observed that shifted in wavenumber as the experimental run progressed. These broad peaks are Mie resonances, which are produced by the weak spontaneous scattering of light across the Raman spectral range [7–10]. An example of this is shown in Figure S2, which shows an image of the intensity recorded across the spectrum over the course of an experimental run, with the experiment progressing from top to bottom. The intensity of the Raman scattering occurs at fixed wavenumbers and is represented by the vertical lines. A series of broader, less intense Mie resonance peaks can be observed moving from right to left throughout the reaction.

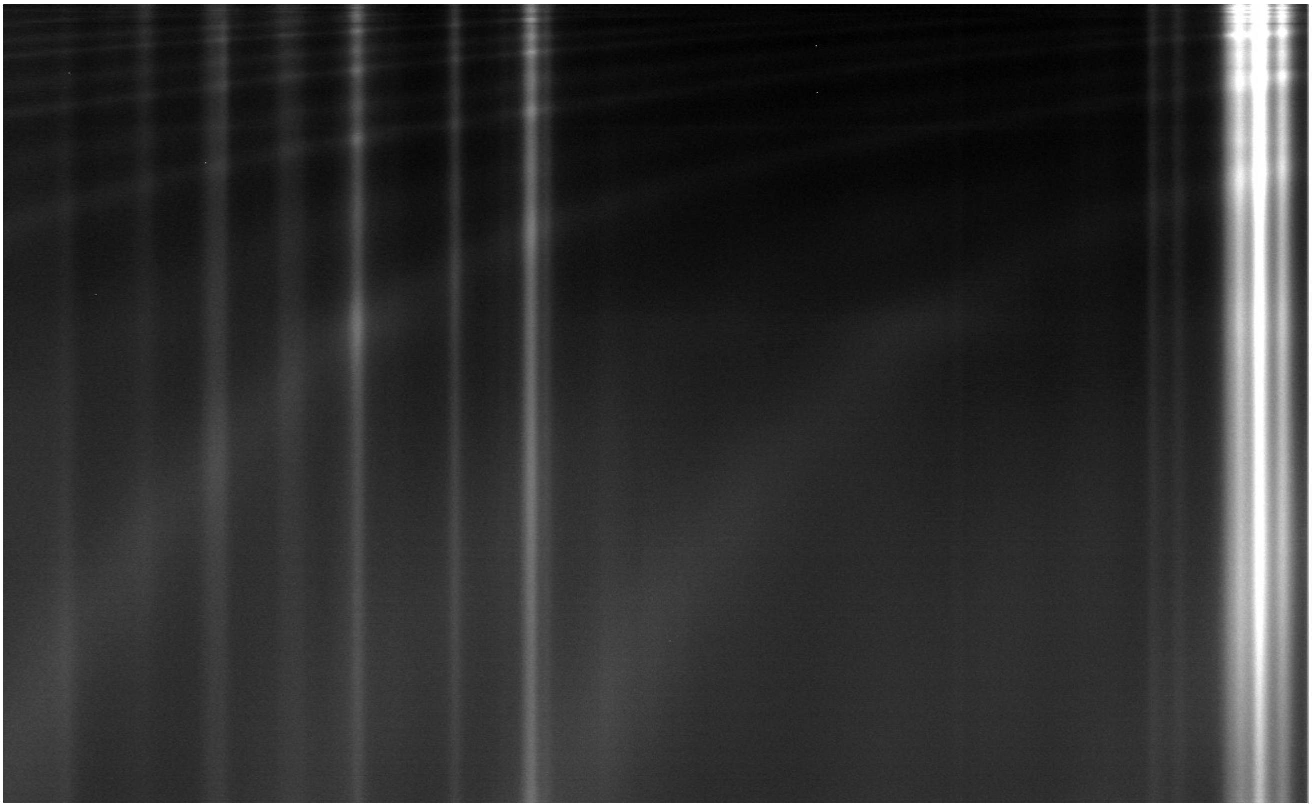

Figure S2: An image showing the intensity of scattered light by an optically trapped droplet of TEOS, in this case undergoing a sol-gel reaction catalysed by NH_4OH. The frame number in the spectra stack increases from top to bottom as the reaction progresses in time, occurring over approximately 70 min. The x-axis is the pixel number and is analogous to the Raman shifts in wavenumbers separated left to right. The intensity is shown by the brightness of each pixel.

The wavelengths of these Mie resonances can be compared to calculated spectra from a theoretical Mie scattering model [11], to retrieve the refractive index and radius of the droplet during optical trapping and therefore throughout the reaction [1,12–16]. However, this analysis requires the Raman signal and Mie resonances to be separated, and so a temporal filtering process was used. Whilst the Raman spectral positions are essentially fixed for these experiments, the Mie spectral positions shift in wavelength over time. For a specified frame, f, in the experimental run, the frames f-n/2 to f+n/2 were chosen using a specified bin width n. The minimum value of the intensity at each pixel across all the spectra in the bin was calculated as the raw Raman signal (Figure S3a). The active frame was then divided by this raw Raman signal and smoothed with a Savitzky–Golay filter to leave a spectrum of wavelength vs. intensity containing only the Mie resonances (Figure S3b). This smoothed experimental Mie spectrum was then compared to calculated Mie spectra to determine the radius and refractive index of the droplet (Figure S3b). As the bin must contain a frame with the raw Raman signal only for each pixel, the choice of bin width is dependent upon the speed at which the Mie resonances shifted through the spectra. As a result, the bin width, n, typically increased over the course of the reaction. A typical experiment using HCl catalysis contained over 2500 frames, and considering each spectrum must be fitted individually, a selected number of frames throughout the experiment were chosen in order to represent the evolution in the size and refractive index of the droplet.

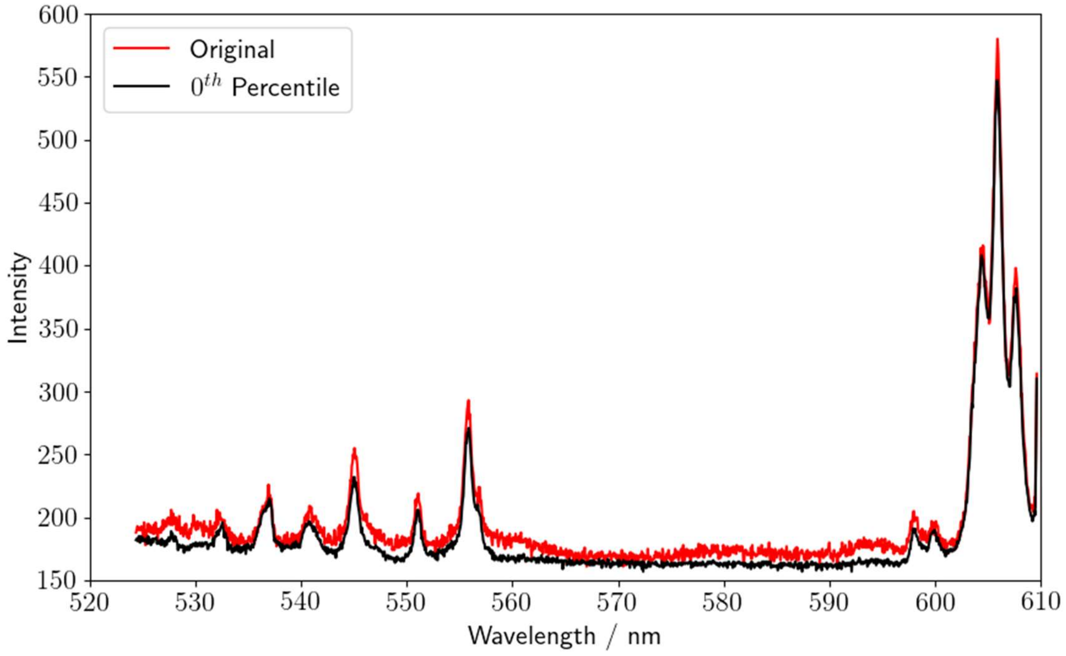

(a)

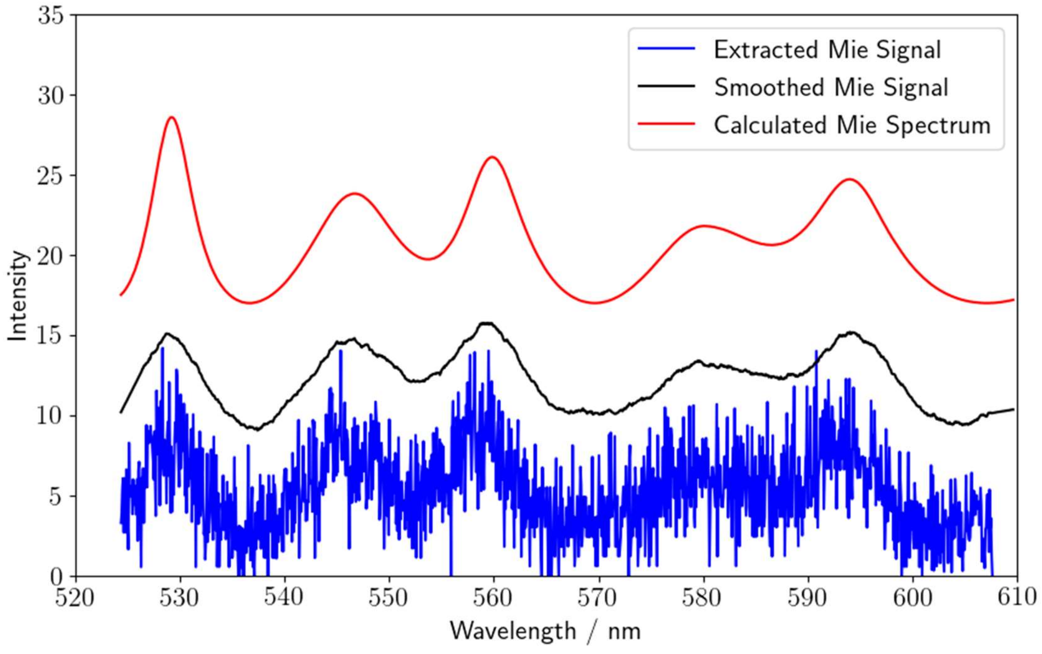

(b)

Figure S3: A demonstration of the process to separate the raw Raman scattering signal and the Mie resonances. (a) A plot showing a representative experimental Raman spectrum in red, containing the sharp, stationary Raman vibrations and the broad, shifting Mie resonances. In black, the raw Raman signal, calculated as the 0th percentile the surrounding 50 frames. (b) In blue, the Mie spectrum extracted from dividing the experimental data by the raw Raman signal (both in (a)), and in black, the experimental spectrum smoothed using a Savitzky–Golay filter. This experimental Mie spectrum was compared to an array of calculated Mie spectra to determine the radius and refractive index of the particle, with the best-fit calculated Mie spectrum shown in red.

MODELLING OF DROPLET VOLUME LOSS

The decrease in droplet volume over time for basic catalysis with NH_4OH was described by two simultaneous first-order equations:

$V - V_{final} = a_1 e^{-k_1 t} + a_2 e^{-k_2 t}$ (1)

Overall, the a_1 and a_2 parameters correspond to the initial volume V_0 for each first-order equation, and the constants k_1 and k_2 describe the rate of change of the droplet volume. To calculate the values of a_1 and k_1, the estimated volume at the end of the initial rapid volume decay – in this case the volume at 58 mins – was subtracted from the volumes from 2 - 13 mins to give a series of

processed volumes that could be modelled with an exponential fit. This enabled k_1 to be directly calculated. For a_2 and k_2, the estimated final volume after the decay was complete was subtracted from the volumes from 58 – 225 mins, with k_2 and a_2 directly taken from an exponential fit of these volumes. The value of a_1 was then calculated as the value of a from the initial exponential fit minus the value of a_2. The subtraction of V_{final} from the modelled volumes accounts for the subtraction of the estimated final volumes. Under acidic conditions with HCl, the volume was still decreasing after at the end of the experiment and it was found that the decrease in volume could be modelled with a single exponential decay. To calculate this, all volumes were processed by subtracting an estimated final volume at the end of the reaction, and the resulting processed volumes were fit to an exponential decay to give the values of a_1 and k_1.

REFERENCES

(1) Shepherd, R. H.; King, M. D.; Marks, A. A.; Brough, N.; Ward, A. D. Determination of the Refractive Index of Insoluble Organic Extracts from Atmospheric Aerosol over the Visible Wavelength Range Using Optical Tweezers. *Atmos. Chem. Phys.* **2018**, *18* (8), 5235–5252. https://doi.org/10.5194/acp-18-5235-2018.

(2) Mitchem, L.; Buajarern, J.; Hopkins, R. J.; Ward, A. D.; Gilham, R. J. J.; Johnston, R. L.; Reid, J. P. Spectroscopy of Growing and Evaporating Water Droplets: Exploring the Variation in Equilibrium Droplet Size with Relative Humidity. *J. Phys. Chem. A* **2006**, *110* (26), 8116–8125. https://doi.org/10.1021/jp061135f.

(3) Fallman, E.; Axner, O. Design for Fully Steerable Dual-Trap Optical Tweezers. *Appl. Opt.* **1997**, *36* (10), 2107–2113. https://doi.org/10.1364/AO.36.002107.

(4) Jones, S. H.; King, M. D.; Ward, A. D. Determining the Unique Refractive Index Properties of Solid Polystyrene Aerosol Using Broadband Mie Scattering from Optically Trapped Beads. *Phys. Chem. Chem. Phys.* **2013**, *15* (47), 20735–20741. https://doi.org/10.1039/c3cp53498g.

(5) Rkiouak, L.; Tang, M. J.; Camp, J. C. J.; McGregor, J.; Watson, I. M.; Cox, R. A.; Kalberer, M.; Ward, A. D.; Pope, F. D. Optical Trapping and Raman Spectroscopy of Solid Particles. *Phys. Chem. Chem. Phys.* **2014**, *16* (23), 11426. https://doi.org/10.1039/c4cp00994k.

(6) Volkert, C. A.; Minor, A. M. Focused Ion Beam Microscopy and Micromachining. *MRS Bull.* **2007**, *32* (5), 389–399. https://doi.org/10.1557/mrs2007.62.

(7) Kiefer, W.; Popp, J.; Lankers, M.; Trunk, M.; Hartmann, I.; Urlaub, E.; Musick, J. Raman-Mie Scattering from Single Laser Trapped Microdroplets. *J. Mol. Struct.* **1997**, *408–409* (96), 113–120. https://doi.org/10.1016/S0022-2860(96)09655-X.

(8) Trunk, M.; Lübben, J. F.; Popp, J.; Schrader, B.; Kiefer, W. Investigation of a Phase Transition in a Single Optically Levitated Microdroplet by Raman–Mie Scattering. *Appl. Opt.* **1997**, *36* (15), 3305. https://doi.org/10.1364/AO.36.003305.

(9) Davies, G.; Driver, J.; Ward, A.; Negahdar, L.; McGregor, J. Operando Studies of Aerosol-Assisted Sol–Gel Catalyst Synthesis via Combined Optical Trapping and Raman Spectroscopy. *J. Phys. Chem. C* **2021**, *125* (41), 22591–22602. https://doi.org/10.1021/acs.jpcc.1c07517.

(10) Hopkins, R. J.; Mitchem, L.; Ward, A. D.; Reid, J. P. Control and Characterisation of a Single Aerosol Droplet in a Single-Beam Gradient-Force Optical Trap. *Phys. Chem. Chem. Phys.* **2004**, *6* (21), 4924–4927. https://doi.org/10.1039/B414459G.

(11) Bohren, C. F.; Huffman, D. R. *Absorption and Scattering of Light by Small Particles*, 1st ed.; Wiley-VCH Verlag GmbH & Co. KGaA: Weinheim, 1983.

(12) Eversole, J. D.; Leung, P. T.; Liu, S. Y.; Lin, H.-B.; Huston, A. L.; Young, K.; Campillo, A. J. High-Precision Identification of Morphology-Dependent Resonances in Optical Processes in Microdroplets. *J. Opt. Soc. Am. B* **1993**, *10* (10), 1955. https://doi.org/10.1364/josab.10.001955.

(13) Ward, A. D.; Zhang, M.; Hunt, O. Broadband Mie Scattering from Optically Levitated Aerosol Droplets Using a White LED. *Opt. Express* **2008**, *16* (21), 16390. https://doi.org/10.1364/OE.16.016390.

(14) Lew, L. J. N.; Ting, M. V.; Preston, T. C. Determining the Size and Refractive Index of Homogeneous Spherical Aerosol Particles Using Mie Resonance Spectroscopy. *Appl. Opt.* **2018**, *57* (16), 4601. https://doi.org/10.1364/AO.57.004601.

(15) Preston, T. C.; Reid, J. P. Determining the Size and Refractive Index of Microspheres Using the Mode Assignments from Mie Resonances. *J. Opt. Soc. Am. A* **2015**, *32* (11), 2210. https://doi.org/10.1364/JOSAA.32.002210.

(16) David, G.; Esat, K. K.; Ritsch, I.; Signorell, R. Ultraviolet Broadband Light Scattering for Optically-Trapped Submicron-Sized Aerosol Particles. *Phys. Chem. Chem. Phys.* **2016**, *18* (7), 5477–5485. https://doi.org/10.1039/c5cp06940h.
